# Benchmarking immunoinformatic repertoire assemblies from bulk RNA-seq and PCR-based V(D)J sequencing using PCR-free SMRT RNA sequencing

**DOI:** 10.1016/j.isci.2026.117564

**Published:** 2026-09-15

**Authors:** Steve Genebrier, Samuel Bastos Serra Trinca, Ophélie Dézé, Marie Cornic, Maiwenn Pineau, Karin Tarte, Michel Cogné

**Affiliations:** 1 UMR1236, Université de Rennes, INSERM, Etablissement Français du Sang, 35000 Rennes, France; 2 SITI Laboratory, Centre Hospitalier Universitaire de Rennes, Etablissement Français du Sang, 35000 Rennes, France

**Keywords:** immune repertoire, deep sequencing, benchmarking

## Abstract

Immune repertoire sequencing (RepSeq) is a crucial immunomonitoring tool, requiring high sensitivity, accuracy, and minimal bias. Although V(D)J mRNA sequencing using 5′ rapid amplification of cDNA ends (RACE-RepSeq) is broadly used, its reliance on PCR may create distortions. Alternatively, *in silico* repertoire-reconstruction (ISRR) tools, computing bulk RNA-seq data, raise concerns regarding accuracy. We compared RACE-RepSeq and ISRR with an advanced PCR-independent approach based on single-molecule real-time (SMRT) sequencing of full-length transcripts. All three approaches applied to immunoglobulin transcripts yielded consistent repertoire-level metrics, including V/J gene usage, CDR3 features, and diversity indices. However, discrepancies emerged in somatic hypermutation assessment, isotype distribution, and at the individual clone level, including incomplete clone overlap and inconsistent isotype assignment, even among abundant clones. These methodological biases exceeded sampling effects. Overall, our results delineate the limitations of each approach in samples from B cell-rich tissues and support SMRT sequencing as a useful reference for immune repertoire benchmarking.

## Highlights

• RACE-RepSeq, *in silico* reconstruction, and SMRT gave concordant metrics in Ig-rich samples
• Clone-level analyses revealed partial overlap and biases in quantification and isotyping
• SMRT sequencing is a robust PCR-independent benchmark for immune repertoire profiling

## Graphical abstract

**Figure:**
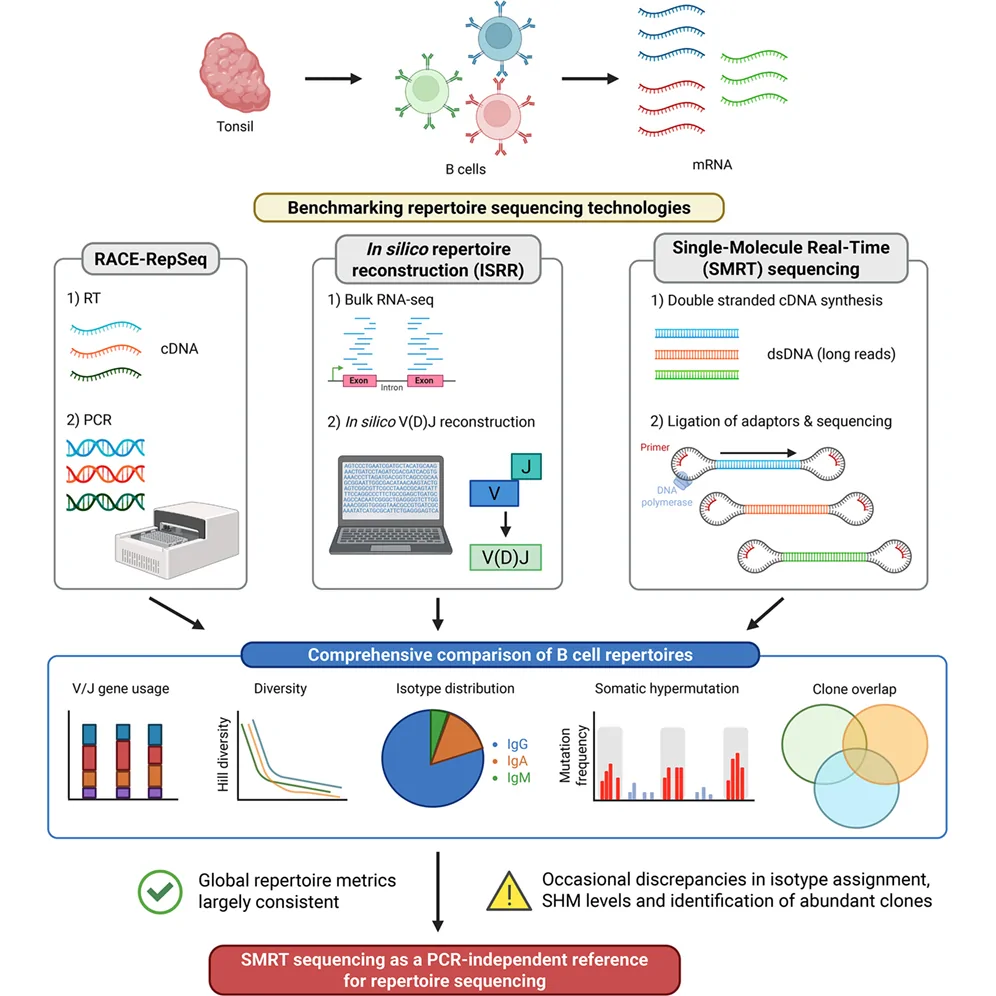

## Teaser

Immunology; Bioinformatics; Transcriptomics

## Introduction

High-throughput sequencing of immune repertoires has profoundly advanced our understanding of adaptive immune responses across diverse contexts, including physiological settings,^1^^,^^2^^,^^3^^,^^4^ infectious and vaccine-induced immunity,^5^^,^^6^ dysimmune disorders,^7^^,^^8^^,^^9^^,^^10^ and malignant hemopathies.^11^^,^^12^^,^^13^^,^^14^ While B cell and T cell repertoires are initially generated using widely similar molecular mechanisms, B-cell repertoire is more complicated to capture due to the secondary introduction of somatic hypermutations (SHM) in immunoglobulin (Ig) variable genes during B cell activation by antigens in secondary lymphoid organs. Moreover, the identification of Ig isotype, a feature associated to the constant (C) region of IGH genes, is of major importance given the variable functional and pharmacological features of the different isotypes.

Various methodologies are now available to profile bulk immune repertoires from tissues, tumor samples, peripheral blood, or sorted lymphoid subsets, with the choice of method primarily dictated by the availability of biological material (tissues, cells, or archived nucleic acids) and specific research objectives.^15^ A critical consideration in repertoire analysis is the choice of starting material: genomic DNA (gDNA) or messenger RNA (mRNA). gDNA-based approaches capture the full theoretical diversity of immune receptors, with a fixed copy number per cell, but do not distinguish expressed from non-expressed receptors and do not cover C regions. They also rely on large primer sets targeting V(D)J sequences, which may fail to amplify alleles carrying single nucleotide polymorphisms (SNPs) or extensive SHM. Conversely, mRNA-based methods selectively profile expressed receptor sequences and enable identification of Ig isotypes, although differences in transcript abundance across lymphocyte subsets may bias clonotype quantification.^15^

Among RNA-based methods, next-generation repertoire sequencing (RepSeq) of V(D)J transcripts using 5′ rapid amplification of cDNA ends (RACE-RepSeq)^16^ is generally regarded as the current gold standard and supports a large fraction of published immune repertoire studies. By anchoring reverse transcription to C genes (e.g., IgH classes, IgL types, and TCR chains), RACE-RepSeq ensures targeted amplification of B cell or T cell receptor transcripts independently of V-region SNPs or SHM. The use of constant-region primers also enables robust isotype determination, and the incorporation of unique molecular identifiers (UMIs) can mitigate PCR amplification biases, a feature only rarely implemented in gDNA-based protocols.^17^^,^^18^ Despite its widespread use and recognized robustness, RACE-RepSeq relies on multiple enzymatic steps, including reverse transcription and PCR amplification, which may introduce distortions in clonotype quantification and sequence fidelity. In particular, the initial reverse transcription step is known to be error prone and limited in processivity,^19^^,^^20^ while PCR amplification may generate jackpot effects that disproportionately inflate the apparent abundance of individual clonotypes. Moreover, RACE-RepSeq libraries often exceed 600 bp in length,^21^ requiring long paired-end sequencing, which increases technical complexity and cost. Despite these limitations, RACE-RepSeq is still widely regarded as the reference RNA-based repertoire method, against which most alternatives approaches are typically benchmarked.

In many experimental and clinical contexts, however, dedicated repertoire sequencing cannot be implemented. Biological material may be limited, or no longer available, as is frequently the case for clinical biopsies and retrospective studies. In such situations, bulk RNA-seq data may already exist, while no targeted repertoire sequencing has been performed. To address this gap, several groups have developed bioinformatics tools capable of reconstructing complete Ig or TCR sequences as contigs through *in silico* assembly of overlapping short reads derived from bulk RNA-seq. Numerous *in silico* repertoire reconstruction (ISRR) tools have been developed, with TRUST4 being the most widely used.^22^ These computational approaches offer a major practical advantage: they enable immune repertoire analysis *a posteriori*, without additional experimental cost or the need for dedicated sample preparation. As a result, ISRR is increasingly used in large-scale clinical and translational studies, where it often constitutes the only feasible strategy to interrogate immune repertoires by reanalyzing existing RNA-seq datasets.^10^ However, these methods must reconstruct full V(D)J sequences from short, overlapping reads derived from multigene families with high sequence homology.^23^^,^^24^ While dominant clonotypes can generally be identified, the accuracy of clonotype reconstruction, quantification, and isotype assignment—particularly for low-frequency or highly similar sequences—remains uncertain. Importantly, the theoretical limitations of ISRR have not been systematically validated against experimental methods that do not rely on PCR amplification.

More recently, long-read RNA-seq technologies based on single-molecule real-time (SMRT) sequencing have emerged as a promising alternative for immune repertoire profiling. By enabling the sequencing of near full-length Ig transcripts, SMRT approaches can theoretically overcome many of the assembly ambiguities inherent to short-read data and provide direct access to constant regions. To date, however, published SMRT-based repertoire studies have retained a PCR amplification step,^25^ which limits their ability to serve as an unbiased reference for evaluating PCR-dependent methods. In addition, the high cost of long-read sequencing has so far restricted its widespread adoption for routine repertoire analysis.

Here, we use PCR-independent SMRT RNA-seq of full-length transcripts to provide a convenient reference for benchmarking RNA-based immune repertoire profiling methods. Using human B cells obtained from tonsils, a widely used secondary lymphoid organ, we compare RACE-RepSeq and TRUST4-based ISRR against SMRT sequencing performed without targeted amplification of Ig transcripts. This experimental design allows us to directly evaluate the extent to which the theoretical limitations of RACE-RepSeq and ISRR translate into practical errors in clonotype identification, quantification, and isotype assignment. By delineating the respective strengths and limitations of these two widely used approaches, our study aims to clarify the reliability of RNA-based immune repertoire analyses across diverse experimental and clinical contexts.

## Results

### Method-related parameters: Expected variations in IGH repertoire recovery

Switched memory B cells were purified from human tonsil samples (*n* = 3), and RNA was extracted to assess the IGH repertoire using three distinct strategies in parallel: RACE-RepSeq, TRUST4-based ISRR based on 2 × 150 bp bulk RNA-seq data, and SMRT long-read sequencing. PCR-independent SMRT sequencing was used here as a reference method, as it directly captures full-length Ig transcripts without targeted amplification.

**Figure 1:**
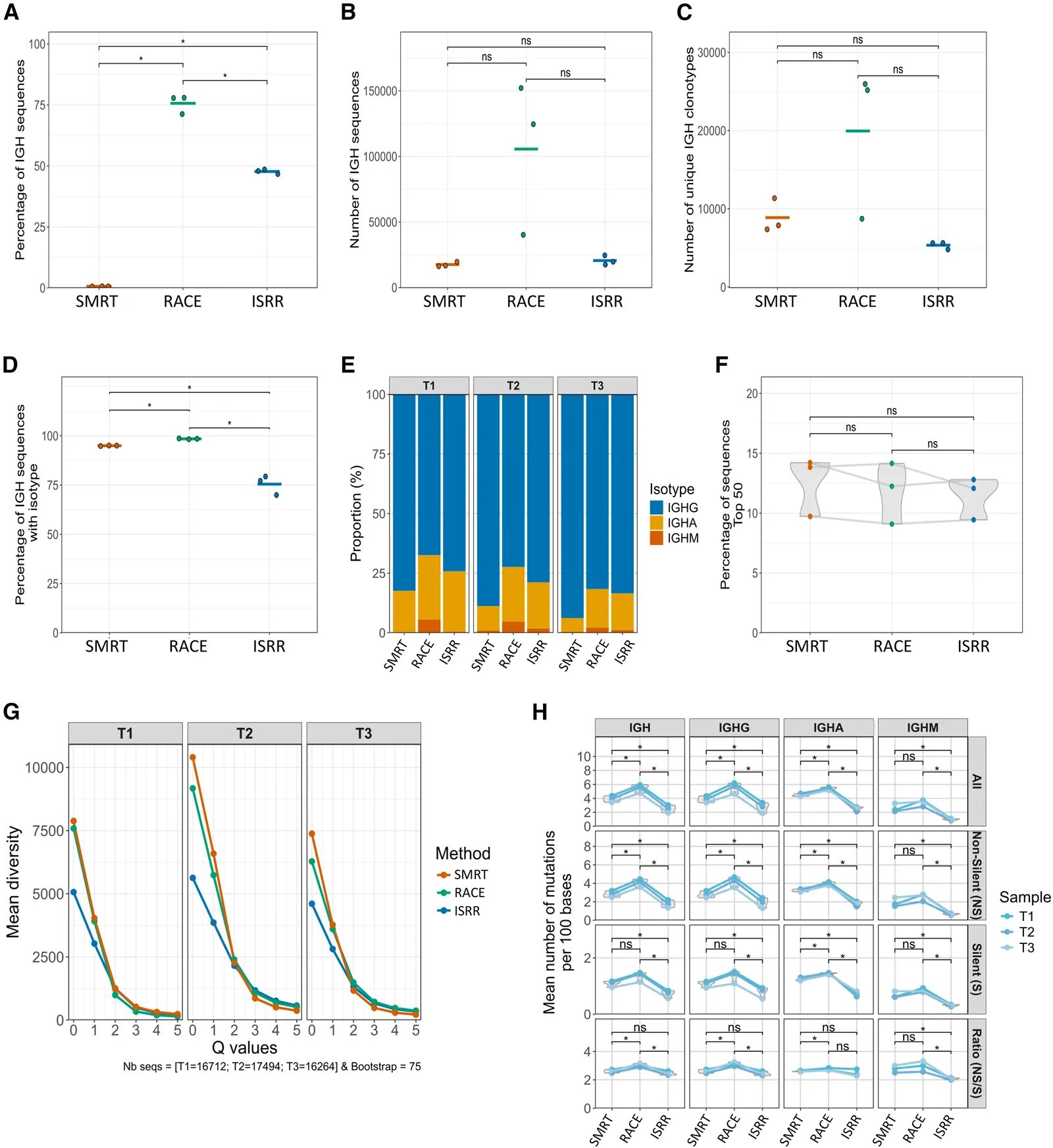
Method-dependent recovery and characterization of the IGH repertoire from human memory B cells (A) Percentage of IGH sequences among total reads obtained with each method. (B) Number of productive IGH sequences recovered. (C) Number of unique clonotypes identified. (D) Percentage of IGH sequences for which an isotype could be assigned. (E) Relative proportions of the major IGH isotypes. (F) Percentage of total IGH sequences accounted for by the 50 most abundant clonotypes (top 50). (G) Hill diversity profiles calculated at the clonotype level, after subsampling, and bootstrap analysis. Points represent the mean values across bootstrap resamples for each *q* value and sample. (H) Somatic hypermutation (SHM) analysis. Points represent the mean values across all sequences within each category and for each sample. Unless otherwise indicated, points represent individual donors (*n* = 3), and bars indicate the mean across donors. Pairwise comparisons in (A)–(D), (F), and (H) were performed using paired Wilcoxon signed-rank tests. One-sided alternatives were RACE > ISRR > SMRT in (A) and RACE > SMRT > ISRR in (D) and (H); all other tests were two sided. Statistical significance was defined as *p* ≤ 0.05 (∗), whereas NS indicates not significant (*p* > 0.05). No inferential statistical tests were performed in (E) or (G). ISRR, *in silico* repertoire reconstruction from bulk RNA-seq data using TRUST4 algorithm; RACE, 5′ rapid amplification of cDNA ends; SMRT, single-molecule real-time sequencing.

All three methods yielded high-quality reads, consistently achieving mean Phred scores above or close to 30. As expected, the proportion of *IGH* transcripts identified by IMGT/HighV-QUEST alignment varied markedly between approaches. SMRT long-read sequencing of the complete transcriptome—performed without targeted reverse transcription or amplification of Ig transcripts—included about 0.6% *IGH* sequences. By contrast, sequence assemblies generated by TRUST4-based ISRR, which specifically reconstructs adaptative immune receptors from bulk RNA-seq data, corresponded to *IGH* sequences in 47% of reads. As anticipated, RACE-RepSeq—utilizing primers specific to Ig constant genes—recovered the highest percentage of *IGH* transcripts (75% of reads) (Figure 1A).

Despite differences in the amount of input RNA (see STAR Methods), the three methods yielded comparable numbers of *IGH* sequences, with a mean of 17,537 for SMRT, 105,627 for RACE-RepSeq, and 20,578 for TRUST4-based ISRR (Figure 1B), as well as unique clonotypes, with a mean of 8,873 for SMRT, 19,950 for RACE-RepSeq, and 5,350 for TRUST4-based ISRR (Figure 1C), even if RACE-RepSeq, which specifically targets Ig mRNAs, sometimes yielded more sequences. When SMRT sequencing was used as a reference, both RACE-RepSeq and TRUST4-based ISRR recovered a similar order of magnitude of clonotypes, indicating that neither approach grossly over- or underestimated repertoire size at the global level.

Using RNA as starting material allows for Ig isotype determination, as mRNA splicing juxtaposes C exons downstream of recombined V(D)J exons. Consequently, the RACE-RepSeq method—with RT anchored on C regions—assigned their isotype in nearly 100% of cases. SMRT long reads also demonstrated strong isotype assignment capability, with about 95% of *IGH* transcripts including VDJ-CH1 spliced junctions. By contrast, TRUST4-based ISRR assemblies were of variable length, with a focus on the CDR3 region and identified a VDJ-associated isotype in only 70%–80% of cases (Figure 1D). Among detected isotypes, the distribution of IGHA, IGHG, and IGHM was broadly consistent across all three methods, with IGHG being the most prevalent (∼80%), followed by IGHA (∼18%) (Figure 1E). As expected with RNA prepared from enriched switched memory B cells, IGHM was rare in all three samples (mean: ∼0.085% for SMRT, ∼4% for RACE-RepSeq, ∼1.11% for TRUST4-based ISRR). However, RACE-RepSeq—which uses cDNA independently extended from IGHM-, IGHG-, and IGHA-specific primers—artifactually yielded the most notable IGHM representation (Figure 1E).

To assess whether methodological biases affected the apparent dominance of expanded clonotypes, we next examined the fraction of the repertoire occupied by the 50 most abundant clonotypes (top 50). This metric was highly comparable across the three methods, with mean values of 12.59% for SMRT, 11.89% for RACE-RepSeq, and 11.43% for TRUST4-based ISRR (Figure 1F), indicating that all approaches similarly capture the degree of clonal expansion and that no method systematically inflates or compresses the contribution of the most frequent clonotypes.

For diversity assessment, the number of unique clonotypes identified by each method was broadly comparable (Figure 1C). Although RACE-RepSeq yielded the highest clonotype count and TRUST4-based ISRR the lowest, the differences remained within a similar range. Hill diversity profiles^26^ generated after subsampling revealed highly comparable diversity across the three methods (Figure 1G). TRUST4-based ISRR showed a slightly reduced richness (Q = 0), reflecting fewer rare clonotypes detected by *in silico* reconstruction, but the corresponding curve rapidly converged with those of SMRT and RACE-RepSeq as Q increased, indicating similar behavior for more abundant clones.

Regarding SHM analysis, RACE-RepSeq detected a higher number of mutations—total, nonsynonymous (NS), and synonymous (S)—than the two other methods. For IgH, the mean number of total mutations per 100 bases was approximately 5.42% for RACE-RepSeq, compared with 3.91% for SMRT and 2.49% for TRUST4-based ISRR (Figure 1H). This may reflect some PCR-induced mutations introduced during amplification. Notably, no UMI-based error correction was applied during bioinformatic processing, as this step considerably reduces the number of sequences available for downstream analyses. In addition, the lower SHM levels observed with TRUST4 can be readily attributed to the generation of consensus sequences during assembly, which reduces mutation diversity within clonotypes. In contrast, SMRT sequencing, which does not rely on PCR amplification, provides a conservative estimate of SHM frequency and thus serves as a useful reference for evaluating mutation-related biases. Despite these quantitative differences in mutation counts, the NS/S ratio (Figure 1H, bottom part of the image)—commonly used as a proxy for affinity maturation, despite its known limitations^27^—was only minimally affected (mean IgH NS/S ratio: 2.61 for SMRT, 3.03 for RACE-RepSeq, and 2.46 for TRUST4-based ISRR).

Collectively, these findings reflect the intrinsic technical specificities of each sequencing approach. Although method-dependent differences were observed for several parameters—including IGH recovery, isotype assignment, and somatic hypermutation—these variations were consistent with the underlying principles of each method and did not result in major distortions of the inferred repertoire composition.

### Repertoire-level parameters: Consistent estimates across methods

**Figure 2:**
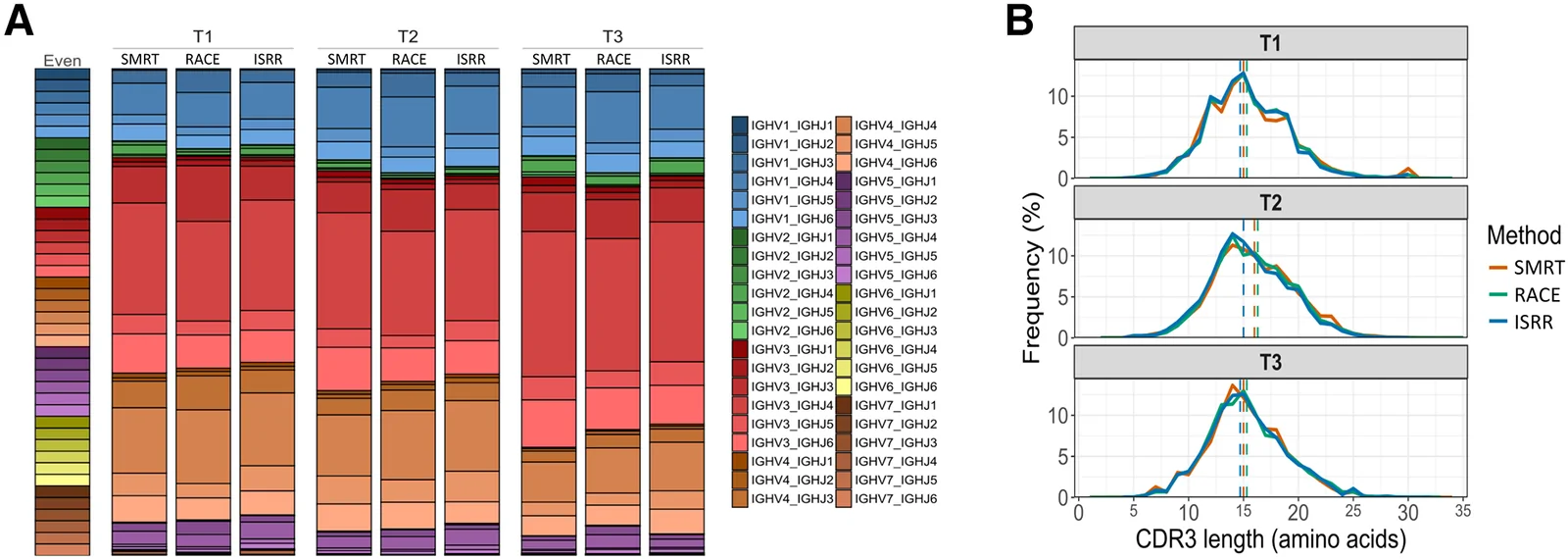
Consistent repertoire-level features across sequencing and reconstruction methods (A) V/J gene usage, shown as stacked bar plots of V-J pairings for each method. (B) Distribution of CDR3 lengths. Medians are displayed as dotted lines. To avoid overlap, a slight offset has been added between methods. ISRR, *in silico* repertoire reconstruction from bulk RNA-seq data using TRUST4 algorithm; RACE, 5′ rapid amplification of cDNA ends; SMRT, single-molecule real-time sequencing.

To compare the results obtained by the three approaches, we examined parameters characterizing the immune response at the global repertoire level. The V/J gene usage was highly similar across methods, as illustrated by the stacked bar plot of V-J pairings (Figure 2A). Notably, the patterns observed with RACE-RepSeq and TRUST4-based ISRR closely matched those obtained by SMRT sequencing, supporting the robustness of these two methods for repertoire-wide analyses. Additionally, the distribution of CDR3 lengths followed an identical Gaussian pattern, with a median length of 15 or 16 amino acids for all three methods (Figure 2B).

In addition, the physicochemical properties of the CDR3 regions, beyond length, as computed using the Alakazam package, were highly similar across methods, as illustrated by radar plots that were largely superimposable between the three approaches (Figure S1).

Taken together, these results demonstrate that both RACE-RepSeq and ISRR approaches provide highly consistent estimates of key repertoire-level parameters when benchmarked against PCR-independent SMRT sequencing and are therefore well suited for describing the overall architecture of the immune repertoires.

### Clone detection: Misestimation errors, even among the most frequent clones

**Figure 3:**
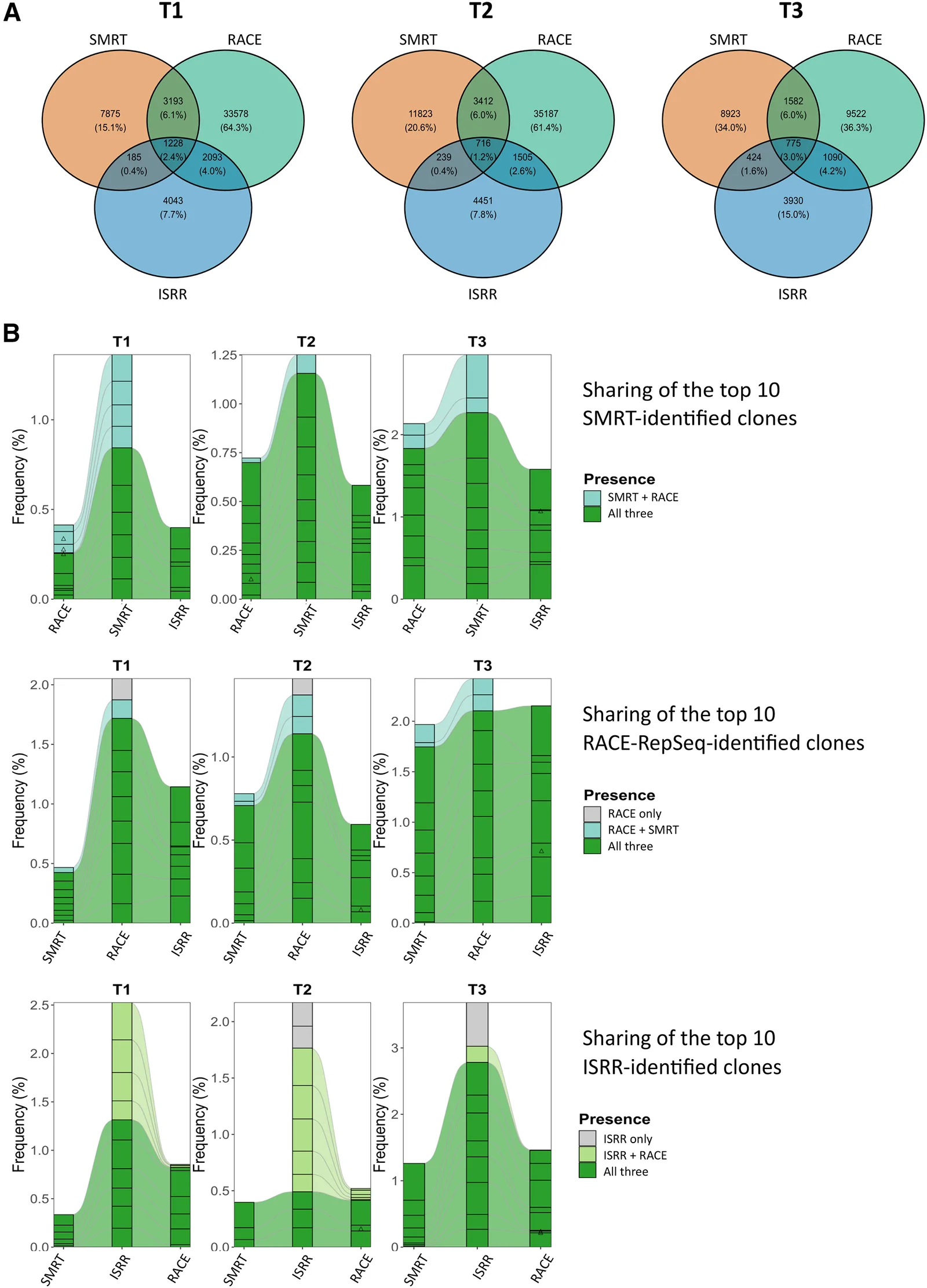
Limited overlap of dominant IGH clones across methods (A) Venn diagrams showing the overlap of global IGH repertoires obtained with the three methods for each sample. (B) Alluvial plots depicting the sharing of the most abundant clones (top 10) across methods. For each part of the image, the reference method is shown in the central column. Discrepancies in isotype assignment are indicated by triangles. ISRR, *in silico* repertoire reconstruction from bulk RNA-seq data using TRUST4 algorithm; RACE, 5′ rapid amplification of cDNA ends; SMRT, single-molecule real-time sequencing.

Repertoire analysis can also enable the tracking of specific clonotypes of interest, such as dominant clonotypes in the context of malignant lymphoid hemopathies,^28^ or public clonotypes—shared sequences between individuals that may indicate convergent immune responses against pathogens.^29^ To assess the ability of each method to identify such clones, we examined the overlap between global repertoires (Figure 3A) and the top 100 most abundant clones detected by each method (Figure S2A). In both cases, clone sharing between any two methods was low (from 4.5% to 14.8% when considering the top 100 clones), consistent with the extreme diversity of the normal human B cell repertoire and the resulting sampling bias.^30^

We then restricted our analysis to the top 10 clones identified by each method and examined their occurrence in the other methods, irrespective of whether they also ranked among the top 10, provided they were detected with at least two copies (Figure 3B). In these analyses, SMRT sequencing was used as the reference to evaluate the consistency of clone detection and quantification by RACE-RepSeq and TRUST4-based ISRR. We deliberately focused on clones rather than clustered clonotypes to avoid potential biases arising from method-specific clustering processes that could create divergent consensus sequences across the three approaches. Overall, we observed a good concordance of the three methods, and even an excellent concordance for sample T3, where 8 out of the top 10 clones were shared by the three methods (dark green color in each part of the image). However, and even for such an analysis restricted to ostensibly abundant sequences, some clones were recovered by only one method (gray) or by two out of the three (turquoise blue or light green). Moreover, even among shared clones, their relative frequencies sometimes differed markedly, as illustrated by the flows connecting the different bars in the alluvial plot. Most notably, when considering the SMRT sequencing-based ranking as a reference that process all transcripts equally, some top 10 clones were undetected by either RACE-RepSeq or TRUST4-based ISRR, while others were underestimated up to ∼30-fold. This effect was especially notable for sequences reconstructed *in silico* by TRUST4: four of the top 10 sequences in sample T1 and five of the top 10 sequences in sample T2 were detected at only minimal frequencies by the RACE-RepSeq method (Figure 3B, bottom part of the image). Such discrepancies were particularly evident when clones identified as frequent by TRUST4 or RACE-RepSeq were compared to their abundance in the SMRT reference dataset, suggesting that methodological biases extend beyond stochastic sampling effects.

Importantly, the three methods were generally concordant with respect to isotype assessment for shared clones, with rare discrepancies being indicated by triangles in Figure 3B. A similar analysis performed on the top 25 clones yielded comparable results (Figure S2B), although isotype discrepancies were more frequent when extending the analysis beyond the most dominant clones, highlighting the advantage of long-read sequencing for reliable constant-region assignment. This extended analysis also confirmed that most highly abundant clones were consistently detected across methods: all top 25 SMRT clones were recovered by at least one of the two other approaches, whereas only five clones in the RACE-RepSeq top 25 list and ten clones in the TRUST4-based ISRR top 25 list lacked confirmation by either of the other methods (Figure S2B).

Since such abundant clones should escape sampling effects, we performed orthogonal validation by targeted amplification and deep sequencing for 10 of them: 5 found only in the RACE-RepSeq top 25 list and 5 found only in the ISSR top 25 list (Table 1). All five RACE-RepSeq clones were readily amplified and confirmed by this approach. Two ranked first in their respective targeted libraries, supporting the quantitative assessment provided by the RACE-RepSeq data. The remaining three ranked between second and fifth, although each was preceded by sequences that were either absent from the RACE-RepSeq top 25 or not detected by RACE-RepSeq. In contrast, the five clones identified exclusively by ISSR as among the top 25 most abundant were not confirmed as abundant by targeted amplification. Instead, they ranked between positions 248 and 4,529 in the analyses of their respective targeted libraries, or in one case could not be successfully amplified (Table 1).

**Table 1:** Orthogonal validation of clones listed among top 25 by only RACE-RepSeq or ISRR

Abbreviated features of the clone tracked by orthogonal validation	Original method	Total sequences obtained	Total unique sequences	Tracked clone			
				Confirmed precise detection	Occurrences	Frequency	Rank
IGHV3-53_J3_AIDDSNNVYAFHI (sample T1)	RACE-RepSeq	99016	11922	yes	1799	1.82%	2
IGHV3-53_J3_ASDYSNYVYAFDV (sample T1)		103199	24214	yes	429	0.42%	5
IGHV4-38-2_J3_ARHEESMAAIRVFDF (sample T1)		91543	9166	yes	3777	4.13%	1
IGHV4-38-2_J1_ARFNGAIAPPG (sample T2)		107534	20977	yes	694	0.65%	4
IGHV4-39_J5_ARVYSGRWFDP (sample T2)		88374	9097	yes	4397	4.98%	1
IGHV4-4_J6_ARVVAADIEGDYGMDV (sample T1)	ISRR	98571	17282	yes	54	0.055%	248
IGHV4-59_J5_AKEGSDYWALDWFDP (sample T1)		15	3	no	0	0.000%	ND
IGHV3-11_J5_ARDGGRNSGWFYTYDS (sample T2)		97858	32299	yes	4	0.004%	4004
IGHV3-11_J1_ARVGIVVEH (sample T3)		99555	7309	yes	1	0.001%	4529
IGHV4-31_J6_ARVASNYGFWSGYRRG DYFYYYMDV (sample T3)		90538	16838	yes	8	0.009%	2178

Selected clonotypes from the top 25 lists identified exclusively by RACE-RepSeq or ISRR were submitted to validation by selective amplification with dedicated primers, followed by individual library construction and deep sequencing. Features of each library, together with frequency and rank of the tracked clone in its relevant library are indicated. (ISRR, *In silico* repertoire reconstruction from bulk RNA-seq data using TRUST4 algorithm; ND, not detected; RACE-RepSeq, 5′ rapid amplification of cDNA ends-repertoire sequencing).

Taken together, these results indicate that while the three approaches broadly capture the same dominant clones, substantial differences remain in clone quantification and ranking. Orthogonal validation further supports the quantitative accuracy of RACE-RepSeq for abundant clones, whereas clones identified as highly expanded exclusively by TRUST4-based ISRR frequently reflect inaccurate abundance estimates.

## Discussion

Ig repertoire analysis provides a powerful mean to assess the quality and dynamics of immune responses in both physiological and pathological contexts. When performed from RNA, it enables access to the expressed Ig transcripts, with RACE-RepSeq used in numerous studies as a standard approach. More recently, ISRR from bulk RNA-seq data has emerged as an attractive alternative, particularly for clinical and translational studies where dedicated repertoire sequencing is often not feasible. TRUST4 has notably become a widely used ISRR tool and has demonstrated its utility in large patient cohorts.^10^^,^^22^

Despite their widespread use, the limitations of both RACE-RepSeq and ISRR have rarely been evaluated experimentally against an independent reference relying neither on PCR amplification nor on predicted sequence assemblies. Long-read sequencing can make this possible by enabling full-length Ig transcript sequencing. To date, however, its cost and the inclusion of an initial PCR step in published SMRT-based repertoire studies^25^ has limited its suitability as an unbiased benchmark for PCR-dependent methods. As it becomes more affordable and broadly available, we now show that PCR-independent SMRT sequencing can efficiently identify and quantify Ig clonotypes with minimal methodological bias. In this study, such a method was leveraged as a useful reference for evaluating the two RNA-based approaches that are mostly used in practice: RACE-RepSeq and ISRR from bulk RNA-seq using TRUST4.

The distinct designs of the three approaches were expected to result in some method-dependent differences. In terms of focus, TRUST4 is dedicated to reconstructing Ig and TCR sequences, to the exclusion of any other transcript, and RACE-RepSeq, which specifically targets IGH transcripts using constant-region primers, likewise yielded predominantly IGH sequences. In contrast, untargeted SMRT sequencing captured only a fraction of reads of IGH origin but within a fully reliable, full-length whole transcriptome dataset. Nevertheless, the total number of IGH sequences obtained and the number of unique clonotypes were remarkably similar across the three approaches.

Our analyses also demonstrate a striking concordance across the three approaches for several major broad characteristics of the IGH repertoire: V and J gene usage, CDR3 length distributions, and physicochemical properties of the CDR3 regions were highly similar, with profiles obtained by RACE-RepSeq and TRUST4-based ISRR closely matching those derived from PCR-independent SMRT sequencing. These results indicate that, despite their different experimental and computational workflows, all three approaches robustly capture the global architecture of the IGH repertoire.

These findings have important practical implications. Many immunological studies—particularly in infectious disease, vaccination, and autoimmunity—aim primarily to characterize repertoire-wide features rather than to track individual clonotypes with high precision. In such contexts, our results support the notion that both RACE-RepSeq and ISRR from bulk RNA-seq provide reliable and largely interchangeable estimates of global repertoire properties, even when benchmarked against a PCR-independent reference.

In contrast to the strong concordance observed at the repertoire-wide level, more precise analyses focusing on individual clonotypes revealed substantial inconsistencies across methods. Overlap between complete repertoires, as well as between the most abundant clonotypes, was limited, in line with previous reports highlighting the impact of sampling effects in highly diverse polyclonal repertoires.^1^^,^^30^^,^^31^^,^^32^ Moreover, even when restricting the analysis to the top-ranked (top 10 or top 25) clones—whose detection should be robust to stochastic sampling—some discrepancies in both presence and relative abundance were observed. Orthogonal validation of clones claimed as abundant by only one of the three methods showed that sequences reported exclusively in the RACE-RepSeq dataset were readily confirmed as among the most prevalent sequences by targeted amplification followed by deep sequencing. By contrast, sequences presented as abundant only by ISRR were detected at low frequencies in the libraries generated by targeted amplification.

Taken together, these results suggest that abundance estimates for some clones may be biased by these two methods but that this risk is lower with RACE-RepSeq than with ISRR. This conclusion is further strengthened by the potential risk of artifactual sequence assembly in the ISRR approach.

These differences most likely reflect the intrinsic focus and bias of each method rather than disparities in sequencing quality, since the MiSeq, NovaSeq, and Revio platforms used for RACE-RepSeq, bulk RNA-seq, and SMRT sequencing, respectively, are all state-of-the-art instruments validated to deliver high-quality sequence data over Q30.

Importantly, the use of PCR-independent SMRT sequencing as a reference suggests that these discrepancies cannot be fully explained by sampling noise alone. Clonotypes identified as highly abundant by RACE-RepSeq or TRUST4-based ISRR were sometimes detected at much lower frequencies, or not at all, in the SMRT dataset, and vice versa. These observations point to methodological biases that affect clonotype quantification and, in some cases, clonotype identification itself. While such biases have long been suspected—PCR jackpot effects for RACE-RepSeq and assembly ambiguities for *in silico* reconstruction—our study provides experimental evidence that they can impact even highly abundant clonotypes. This has direct implications for applications that rely on accurate clonotype-level information, such as minimal residual disease monitoring, tracking of dominant clones in lymphoid malignancies, or the identification of public clonotypes associated to specific conditions.

Beyond the variations in precise clonotype identification and quantification, two additional and more global biases of RACE-RepSeq and ISRR were noticed. The first concerns isotype assignment, which, although largely concordant for the most abundant clonotypes, differed in efficiency and showed an overall bias across the three methods. Because isotype assignment is a key immunomonitoring parameter for infection- or vaccine-induced responses, these differences are non-trivial. Nearly all IGH transcripts recovered by SMRT carried an identifiable and reliable isotype, owing to their full-length coverage. By contrast, TRUST4 reconstructed sequences were long enough for isotype calling in only ∼75% of cases, which nevertheless is a good performance for an ISRR pipeline. RACE-RepSeq warrants specific consideration in this context. Anchoring the initial cDNA synthesis and PCR steps on the IGH C region ensures that most sequences carry an isotype identifier, as with SMRT sequencing. However, the method requires three independent cDNA and PCR reactions whose efficiency to amplify μ, γ, and α transcripts inevitably differs, even when multiplexed. This bias was particularly evident in our switched memory B cell samples, in which few functional IGHM transcripts are expected and are evidenced by both SMRT sequencing and ISRR, yet their relative abundance appears artificially inflated by RACE-RepSeq.

A last global bias of RACE-RepSeq in our study is a trend to over-estimate the SHM level, likely due to PCR errors, whereas TRUST4 may by contrast underestimate SHM due to the use of consensus sequences during *in silico* assembly. In this context, SMRT sequencing again provides a conservative estimate of mutation frequencies and serves as a useful reference for evaluating mutation-related biases. Overall, such method-dependent differences are fully consistent with the underlying technical principles of each approach and do not appear as major distortions in repertoire characterization.

Although we benchmarked state-of-the art methods routinely used by multiple teams, there is certainly room for improvement of these methods. A more systematic use of UMIs in RACE-RepSeq would notably help limit SHM overestimation, albeit at the cost of lower repertoire depth.

For ISRR, several bio-informatic tools are available to infer immune repertoires from RNA-seq data, such as MiXCR,^33^ Vidjil,^34^ V’DJer,^35^ ImReP,^36^ but TRUST4 is the most recent, is the most widely used and is considered the most accurate.^22^ Importantly, *in silico* reconstruction tools such as TRUST4 play an indispensable role in clinical and retrospective studies, where only low-quality RNA samples or previously obtained bulk RNA-seq data are available and strictly limit the strategies able to provide information about immune repertoires.

Our results should therefore not be viewed as questioning the utility of these strategies, but rather as defining the boundaries within which their outputs can be interpreted with confidence. As long-read sequencing technologies continue to decrease in cost and increase in throughput, PCR-independent SMRT sequencing may already serve as a benchmark or validation tool for repertoire analyses, although a minimal risk of errors remains for any method using reverse transcription, including SMRT sequencing. Further advances in cost, efficiency, and depth will be needed before an unbiased approach can realistically and routinely replace existing repertoire profiling methods.

In summary, despite the limited sample size, our study indicates that RACE-RepSeq and ISRR from bulk RNA-seq provide highly consistent and reliable estimates of global IGH repertoire features, even when evaluated against a PCR-independent reference. By contrast, clone-level analyses reveal methodological biases that can affect the identification and quantification of individual clones, including highly abundant ones. By leveraging PCR-independent SMRT sequencing as a reference, our work delineates the respective strengths and limitations of widely used RNA-based repertoire profiling strategies and provides a framework for their informed application in both research and clinical settings. However, these conclusions should not be overgeneralized, as they were drawn from samples in which Ig transcripts are abundant and readily accessible; in more complex biological or clinical settings, method choice may be more constrained. In such situations, the main takeaway may be the overall agreement of the three methods compared.

### Limitations of the study

Several limitations of our study should be acknowledged. First, while we based our comparisons on thousands of IGH sequences characterized for each RNA sample, the number of replicated experiments was limited (*n* = 3), reflecting the methodological nature of the work rather than an attempt at population-level inference. Statistical analyses of these 3 independent experiments were therefore intentionally conservative and descriptive. Second, although PCR-independent SMRT sequencing offers a robust reference, it remains constrained by the need of high-quality RNA and the cost of sequencing, in particular in non B cell enriched samples where deeper sequencing would be required to capture enough Ig reads. These constraints currently limit the applicability of SMRT sequencing as a routine repertoire profiling tool.

## Resource availability

### Lead contact

Further information and requests for resources and reagents should be directed to and will be fulfilled by the lead contact, Michel Cogné (michel.cogne@inserm.fr).

### Materials availability

This study did not generate new unique reagents.

### Data and code availability

• Raw sequencing data generated in this study have been deposited in the NCBI Gene Expression Omnibus (GEO, https://www.ncbi.nlm.nih.gov/geo/) and are available under the following accession numbers: GEO: GSE326979 for 2 × 150 bp RNA-seq data, GEO: GSE326988 for PacBio SMRT sequencing data, and GEO: GSE326977 for RACE-RepSeq data.
• Clonotype abundance tables have been deposited in Zenodo and are publicly available at https://doi.org/10.5281/zenodo.18921756.
• This paper does not report original code.
• Any additional information required to reanalyze the data reported in this paper is available from the lead contact upon request.

## Acknowledgments

We thank the team from Clinique de la Sagesse, Rennes, France for access to tonsil samples. This work was supported by the grant CSR-DEV ANR-24-CE15-3761 from 10.13039/501100001665Agence Nationale de la Recherche, France, to M. Cogne.

## Author contributions

M. Cogne, conceptualization, methodology, funding, validation, supervision, writing – original draft, and writing – review and editing; S.G., investigation, data curation, formal analysis acquisition, and writing – original draft; S.B.S.T., visualization, data curation, and formal analysis acquisition; O.D., methodology and investigation; M. Cornic, methodology and investigation; M.P., data curation; K.T., project administration resources. All authors participated in the discussion and helped revise the manuscripts.

## Declaration of interests

The authors declare no competing interests.

## STAR★Methods

### Key resources table

**Table:** 

REAGENT or RESOURCE	SOURCE	IDENTIFIER
**Biological samples**		

Human palatine tonsil specimens from routine tonsillectomies (*n* = 3)	Clinique de la Sagesse (Rennes, France)	N/A

**Chemicals, peptides, and recombinant proteins**		

TRIzol Reagent	Invitrogen	Cat# 15596026
SuperScript IV Reverse Transcriptase	Invitrogen	Cat# 18090050
Uracil-DNA glycosylase	New England Biolabs	Cat# M0280S
Phusion High-Fidelity DNA Polymerase	New England Biolabs	Cat# M0530S
AMPure XP beads	Beckman Coulter	Cat# A63881

**Critical commercial assays**		

Switched Memory B Cell Isolation Kit, human	Miltenyi Biotec	Cat# 130–093-617
RNA ScreenTape	Agilent Technologies	Cat# 5067–5576
NucleoSpin Gel and PCR Clean-up	Macherey-Nagel	Cat# 740609.50
PippinHT cassette 1.5% agarose 300 pb–1.5 kb	Sage Science	Cat# HTC1510
D1000 ScreenTape	Agilent Technologies	Cat# 5067–5582
MiSeq Reagent Kit v3 (600 cycles)	Illumina	Cat# MS-102–3003
TruSeq Stranded mRNA Library Prep kit	Illumina	Cat# 20020594
SMRTbell Express Template prep iso-seq kit	Pacific Biosciences	Cat# 100–938-900
QIAGEN Multiplex PCR kit	QIAGEN	Cat# 206143
Qubit dsDNA BR Assay kit	ThermoFisher	Cat# Q32853

**Deposited data**		

Raw sequencing data	This paper	Gene Expression Omnibus (GEO) accession numbers: 2 × 150 bp RNA-seq data, GEO: GSE326979; PacBio SMRT sequencing data, GEO: GSE326988; RACE-RepSeq data, GEO: GSE326977
Clonotype abundance table	This paper	Zenodo repository https://doi.org/10.5281/zenodo.18921756

**Oligonucleotides**		

Primers for RACE-RepSeq library, see Table S1	Pascal et al.^6^ This paper	N/A
Primers for orthogonal validation, see Table S3	This paper	N/A

**Software and algorithms**		

R v4.2.3	The R Foundation^37^	https://www.r-project.org/
TRUST4 v1.0.5.1	Song et al.^22^	https://github.com/liulab-dfci/TRUST4
MIGEC v1.2.9	Shugay et al.^38^	https://github.com/mikessh/migec
FLASH v1.2.11	Magoč et al.^39^	https://github.com/ebiggers/flash
IMGT HighV-Quest	The international ImMunoGeneTics information system. Alamyar et al.^40^	IMGT/V-QUEST reference directory release 202430-2 (Human IG) https://www.imgt.org/IMGTindex/IMGTHighV-QUEST.php
SCOPer v1.3.0	Gupta et al. t^41^	https://cran.r-project.org/package=scoper
Immunarch v0.9.1	Nazarov et al.^42^	https://cran.r-project.org/package=immunarch
Alakazam v1.3.0	Gupta et al.^43^	https://cran.r-project.org/package=alakazam

### Experimental model and study participant details

#### Human participants and tonsil samples

Human palatine tonsils were obtained from patients undergoing routine tonsillectomy at Clinique de la Sagesse (Rennes, France). Fresh surgical specimens were collected immediately after surgery and processed for research purposes. Tonsil samples from all three participants were analyzed using each of the three sequencing approaches; therefore, samples were not allocated to separate experimental groups. Information regarding participant age and sex was not available for this study.

#### Ethics statement

Collection and storage of human samples were performed under the Rennes University Hospital agreement DC-2015–2565. Written informed consent was obtained from all participants prior to sample collection. The study was approved by the French Ministry of Higher Education and Research and conducted in accordance with the principles of the Declaration of Helsinki.

### Method details

#### Purification of tonsil B cells

Fresh tonsil specimens were cut into approximately 3 mm^3^ fragments and perfused with culture medium. Mononuclear cells were isolated by density gradient centrifugation. Switched memory B cells were subsequently enriched by negative magnetic selection using the human Switched Memory B Cell Isolation Kit (Miltenyi Biotec), according to the manufacturer’s instructions. Cell separation was performed on an AutoMACS Pro Separator using the depletes program.

#### RNA extraction

Total RNA was extracted from purified B cells using TRIzol reagent according to the manufacturer’s instructions. Briefly, cells were lysed in 1 mL of TRIzol, followed by phase separation with 200 μL chloroform and RNA precipitation with 0.5 mL isopropanol per milliliter of initial TRIzol. RNA pellets were washed with 80% ethanol, air-dried, and resuspended in 20 μL nuclease-free water. RNA concentration and purity were assessed using a NanoDrop spectrophotometer (DeNovix) by measuring the A260/A280 and A260/A230 absorbance ratios. RNA integrity was evaluated using a 4200 TapeStation system (Agilent), and only samples with an RNA Integrity Number (RIN) greater than 7 were further used.

#### RACE-RepSeq

For each sample, 500 ng of total RNA was reverse-transcribed using SuperScript IV Reverse Transcriptase (Invitrogen) with a cap RACE primer containing a unique molecular identifier (UMI) and consensus reverse primers specific for the immunoglobulin heavy chain constant regions (Cμ, Cγ, and Cα; see Table S1). cDNA was treated with U-DNA Glycosylase (New England Biolabs) and purified using NucleoSpin Gel and PCR Clean-up columns (Macherey-Nagel) according to the manufacturer’s instructions.

Library was generated by amplifying 5 μL of purified cDNA using Phusion High-Fidelity DNA Polymerase (New England Biolabs) and nested reverse primers targeting the CH1 exons of Cμ, Cγ, and Cα (Table S1). PCR cycling conditions consisted of an initial denaturation at 95°C for 2 min, followed by 25 cycles of 95°C for 10 s, 62°C for 30 s, and 72°C for 45 s, with a final extension at 72°C for 5 min. PCR products were purified using AMPure XP beads (Beckman Coulter) at a 0.6× bead-to-sample ratio and eluted in 30 μL of elution buffer. Purified amplicons (5 μL) were subsequently indexed with Illumina adapters by PCR using Phusion High-Fidelity DNA Polymerase under the following conditions: 95°C for 30 s, followed by 11 cycles of 95°C for 10 s, 62°C for 30 s, and 72°C for 45 s, with a final extension at 72°C for 5 min. Indexed PCR products were purified using AMPure XP beads (0.6× bead-to-sample ratio), quantified using a TapeStation system (Agilent Technologies), pooled equimolarly, and size-selected for fragments ranging from 600 to 1,000 bp using a PippinHT system (Sage Science) with a 1.5% agarose cassette. The final library was assessed for quality using a D1000 ScreenTape assay on a 4200 TapeStation system (Agilent Technologies) before paired-end sequencing (2 × 300 bp) on an Illumina MiSeq instrument using a custom sequencing primer (Table S1).

Basic description of the datasets generated by RACE-RepSeq is provided in Table S2.

#### RNA sequencing

For short-read RNA sequencing, 1,200 ng of total RNA was used for poly(A)+ RNA enrichment and library preparation with the TruSeq Stranded mRNA Library Prep Kit (Illumina) according to the manufacturer’s instructions. Paired-end sequencing (2 × 150 bp) was performed on an Illumina NovaSeq platform, generating approximately 15 Gb of sequence data per sample.

Basic description of the datasets generated by short-read RNAseq is provided in Table S2.

#### Single-molecule real-time RNA sequencing

For long-read transcriptome sequencing, 1,500 ng of total RNA was used for full-length cDNA library preparation with the SMRTbell library preparation kit for Iso-Seq (Pacific Biosciences) according to the manufacturer’s instructions. Libraries were sequenced on a PacBio Revio system by Macrogen, generating approximately 10.5 Gb of sequence data per sample.

Basic description of the datasets generated by SMRT sequencing is provided in Table S2.

#### Orthogonal validation of selected clonotypes

Selected clonotypes listed in the Top 25 lists by only RACE-RepSeq or ISRR were submitted to orthogonal validation through selective amplification and deep sequencing.

Reverse transcription of 500 ng of total RNA into cDNA was done using SuperScript IV (Invitrogen). The resulting cDNA was treated with U-DNA-Glycosylase (New England Biolabs) and purified using NucleoSpin Gel and PCR Clean-Up columns (Macherey-Nagel). For each targeted PCR reaction, one microliter of cDNA was used as template and amplified using Phusion High-Fidelity DNA Polymerase (New England Biolabs). The primers designed for each tracked amplicon included a specific VH forward primer overlapping the CDR1 region and an appropriate reverse primer targeting the CH1 exon of the Cγ or Cα genes (primer sequences are provided in Table S3). Cycling conditions were as follows: 2 min at 95°C; 35 cycles of 10 s at 95°C, 30 s at 60°C, and 45 s at 72°C; followed by a final extension of 5 min at 72°C. PCR products were purified using AMPure XP beads (Beckman Coulter) at a 0.6× reaction volume ratio and resuspended in 30 μL of NEB elution buffer (Qiagen). PCR products were purified using the NucleoSpin Gel and PCR Clean-up kit (Macherey-Nagel) according to the manufacturer’s instructions. Amplicon quality was assessed using the D1000 ScreenTape assay on a TapeStation system (Agilent Technologies), and DNA concentrations were determined using a Qubit fluorometer (Thermo Fisher Scientific). Subsequently, purified amplicons adjusted to 20 ng/μL were sent for NGS library synthesis and ultra-deep MiSeq paired-end sequencing to the Genewiz/Azenta platform.

Sequence alignment and V(D)J gene assignment were performed using IMGT/HighV-QUEST 40 with the human IG reference database (release no. 202614-2). For each library, the exact sequence corresponding to the tracked clone (provided in Table S4) was ranked according to its abundance among all the aligned V(D)J sequences observed.

#### BCR repertoire reconstruction from RNA sequencing

B cell receptor (BCR) repertoires were reconstructed from bulk RNA sequencing data using TRUST4 (see key resources table). Raw FASTQ files were processed directly with default parameters to assemble productive V(D)J rearrangements and reconstruct immunoglobulin heavy-chain sequences.

#### RACE-RepSeq data processing

RACE-RepSeq reads were processed using MIGEC for unique molecular identifier (UMI)-based read grouping and error correction (see key resources table). UMI groups supported by at least one read (MIG = 1) were retained. Paired-end reads were merged using FLASH (see key resources table) with a minimum overlap of 30 bp and a maximum overlap of 300 bp. Read pairs that could not be merged were concatenated with undefined nucleotides (N) inserted between forward and reverse reads.

#### Sequence alignment and clonotype assignment

For RACE-RepSeq and single-molecule real-time (SMRT) sequencing datasets, V(D)J gene assignment was performed using IMGT/HighV-QUEST with the human immunoglobulin reference database (see key resources table). Only productive rearrangements with identified V and J genes and a defined CDR-H3 region were retained for downstream analyses.

Clonotype assignment was performed using the hierarchicalClones function implemented in SCOPer (see key resources table), based on shared IGHV gene usage, shared IGHJ gene usage, identical junction length, and junction sequence similarity. The junction distance threshold was automatically inferred using SCOPer’s threshold inference procedure. Each clonotype was represented by the most abundant junction nucleotide sequence within the clone. Both clone-level and clonotype-level datasets were retained for downstream analyses. Immunoglobulin isotypes and subclasses were assigned according to constant region gene annotation and confirmed by matching isotype-specific primer sequences.

#### Repertoire analysis

Repertoire diversity was assessed by calculating Hill diversity profiles at the clonotype-level using immunarch (see key resources table). To account for differences in sequencing depth, repertoires were randomly subsampled to the size of the smallest sample prior to diversity analyses. Bootstrap iterations were optimized for each analysis to ensure stable diversity estimates.

Physicochemical properties of CDR-H3 sequences were calculated using Alakazam (see key resources table). Within each sample, sequences obtained with the three methods were pooled, and each physicochemical feature was *Z* score standardized using the R scale() function. The median standardized value of each feature was then calculated separately for each method. These method-specific medians were min–max rescaled to a 0–1 range within each sample and visualized as radar plots using the fmsb package.^44^

Somatic hypermutation (SHM) frequency was calculated as the number of nucleotide mutations per 100 aligned IGHV nucleotides. For RACE-RepSeq and SMRT datasets, mutation counts relative to the corresponding germline IGHV sequence were extracted from IMGT/HighV-QUEST outputs. For RNA sequencing-derived repertoires, reconstructed TRUST4 sequences were reanalyzed with IMGT/HighV-QUEST to determine mutation frequencies. Because TRUST4 reconstructs consensus contigs from multiple reads, SHM frequencies obtained from RNA sequencing data may underestimate the true mutation burden.

#### Data visualization

Data visualization was performed in R using ggplot2 and associated extension packages.^45^ Shared clones were visualized using Venn diagrams generated with ggvenn.^46^ Alluvial plots illustrating the distribution of the most abundant clones across sequencing methods were generated using ggalluvial.^47^

### Quantification and statistical analysis

#### Statistical analysis

Bioinformatic analyses and statistical tests were performed in R (see key resources table). Statistical comparisons between sequencing methods were performed using the Wilcoxon rank-sum test for unpaired samples. Two-sided tests were used when no directional hypothesis was specified *a priori*, whereas one-sided tests were used when a directional hypothesis was specified *a priori* based on the expected direction of the difference between methods. The statistical test and, where applicable, the direction of the alternative hypothesis used for each comparison are indicated in the corresponding figure legend. Statistical annotations were generated using ggpubr.^48^ Statistical significance was defined as *p* ≤ 0.05 (∗), and *p* > 0.05 was considered not significant (NS).

## Supplemental information

Document S1. Figures S1 and S2, Tables S1–S4, and reference
