# Supplementary material for: Benchmarking immunoinformatic repertoire assemblies from bulk RNA-seq and PCR-based V(D)J sequencing using PCR-free SMRT RNA sequencing
Source: iScience. 2026 Sep 15;29(10):117564. doi: 10.1016/j.isci.2026.117564 (PMC13595170; doi:10.1016/j.isci.2026.117564)
Supplement: Supplementary file 1 [file mmc1.pdf]

**iScience, Volume *29***

## Supplemental information

# Benchmarking immunoinformatic repertoire assemblies from bulk RNA-seq and PCR-based V(D)J sequencing using PCR-free SMRT RNA sequencing

**Steve Genebrier, Samuel Bastos Serra Trinca, Ophélie Dézé, Marie Cornic, Maiwenn Pineau, Karin Tarte, and Michel Cogné**

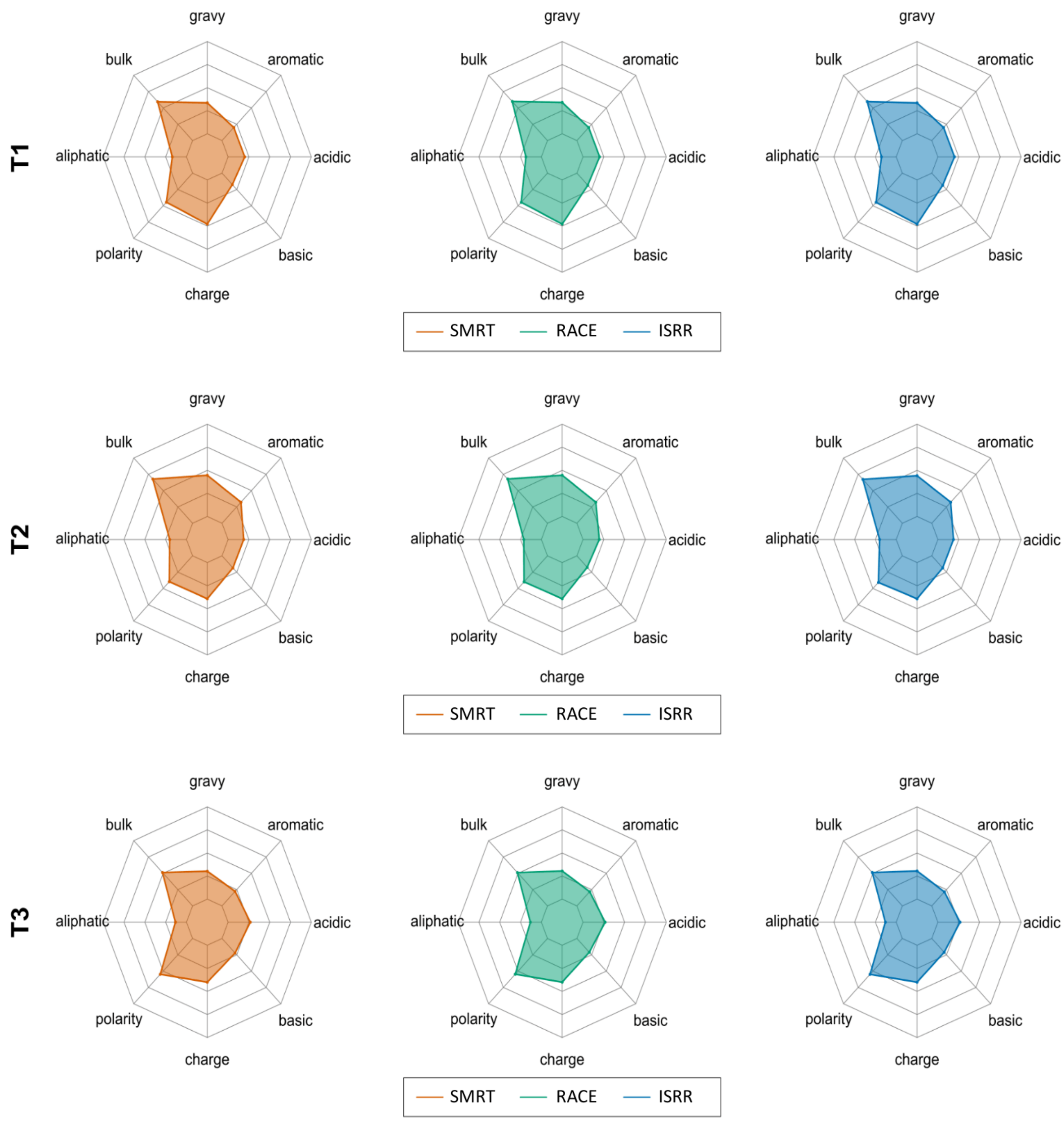


**Supplementary Figure 1. Similar physicochemical properties of CDR3 regions across methods**
Radar plots showing physicochemical properties of CDR-H3 regions computed using the Alakazam package. Values represent median z-score-normalized feature values for each sample and sequencing method, with each axis scaled from 0 to 1 based on the minimum and maximum values observed across the three methods. Profiles are highly similar across the three methods.

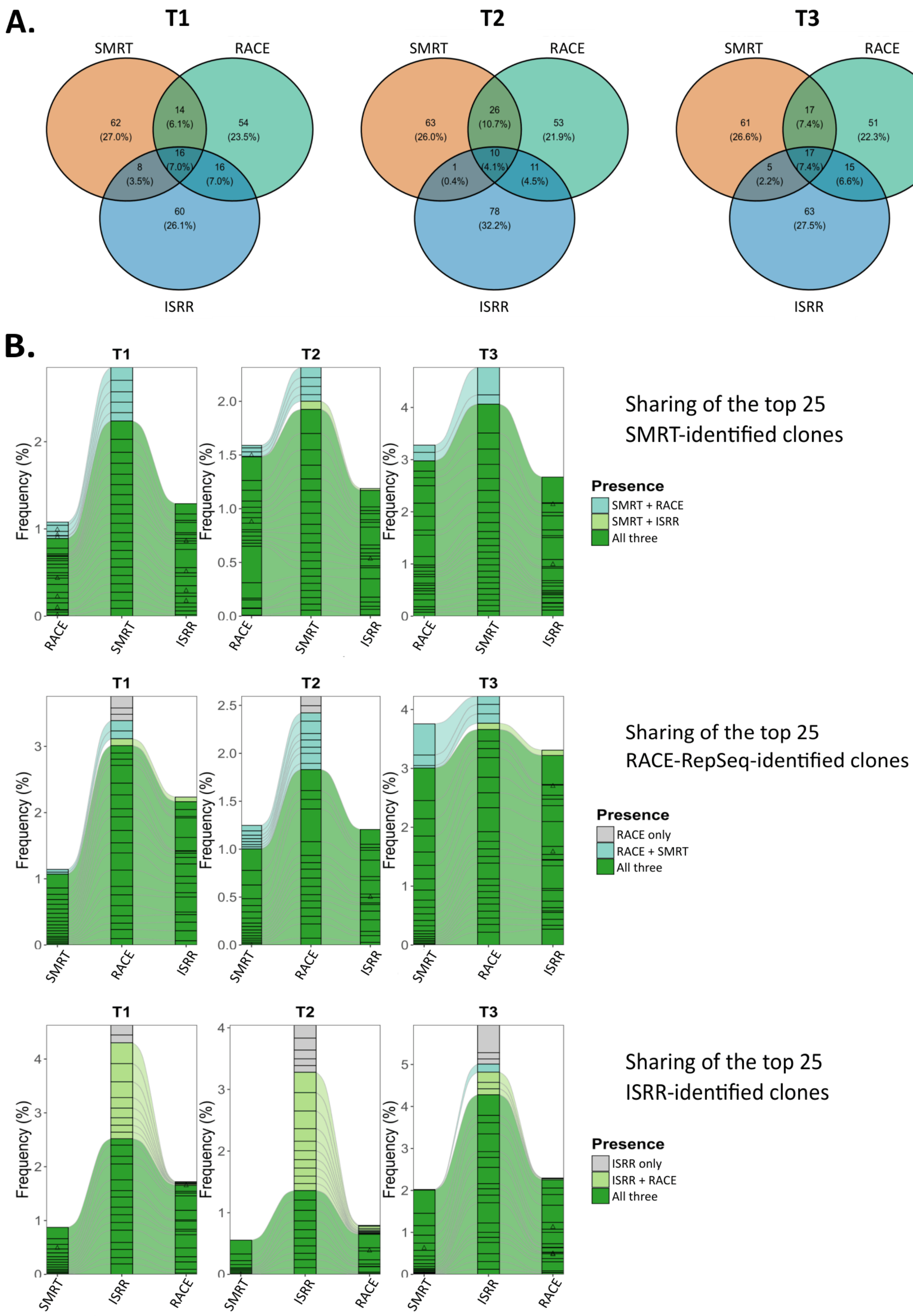


**Supplementary Figure 2. Overlap and isotype concordance of abundant IGH sequences**

(A) Overlap of the Top 100 clones identified by each method.

(B) Alluvial plots depicting the sharing of the most abundant clones (Top 25) across methods. For each panel, the reference method is shown in the central column. Discrepancies in isotype assignment are indicated by triangles.

**Table S1**

**Primers used for RACE-RepSeq, related to STAR Methods. Most of these primers were described in Pascal et al, 2023[1].**

| Primer | Application | Sequence (5' to 3') |
|---|---|---|
| **Reverse transcription** | | |
| UMI-CAP | 5' template switch adaptor mix | AGGCAGUGGTGUGACCGCAGAGUNNNNUNNNNUNNNNUCTT(GGGG) |
| UMI-CAP+4 | | AGGCAGUGGTGUGACCGCAGAGUNNNNUNNNNUNNNNUCTTAAAA(GGGG) |
| Cμ RT CH1 | IgM heavy-chain cDNA synthesis primer | GTGATGGAGTCGGGAAGGAAG |
| Cγ RT CH1 | IgG heavy-chain cDNA synthesis primer | GCCGGGAAGGTGTGCACG |
| Cα RT CH1 | IgA heavy-chain cDNA synthesis primer | GGAAGTTTCTGGCGGTCACG |
| **First PCR amplification** | | |
| UMI-Long Forward | Step-out primer mix anneals on the switch adaptor with Illumina (i5) adapters | TCGTCGGCAGCGTCAGATGTGTATAAGAGACAGGACTAGGCAGTGGTGTGACCGCAGAGT |
| UMI-Short Forward | | TCGTCGGCAGCGTCAGATGTGTATAAGAGACAGGACT |
| Cμ PCR1 nested | IgM heavy-chain cDNA nested primer – (i7) adapter | GTCTCGTGGGCTCGGAGATGTGTATAAGAGACAGAGGGGGAAAAGGGTTG |
| Cγ PCR1 nested | IgG heavy-chain cDNA nested primer – (i7) adapter | GTCTCGTGGGCTCGGAGATGTGTATAAGAGACAGGAAGTAGTCCTTGACCAGGCA |
| Cα PCR1 nested | IgA heavy-chain cDNA nested primer – (i7) adapter | GTCTCGTGGGCTCGGAGATGTGTATAAGAGACAGGCGAYGACCACGTTCCCATCT |
| **Second PCR amplification** | | |
| Illumina Forward 1 | Illumina Index 2 (i5=S513) adapter | AATGATACGGCGACCACCGAGATCTACACTCGACTAGTCGTCGGCAGCGTC |
| Illumina Forward 3 | Illumina Index 2 (i5=S516) adapter | AATGATACGGCGACCACCGAGATCTACACCCTAGAGTTCGTCGGCAGCGTC |
| Illumina Forward 5 | Illumina Index 2 (i5=S518) adapter | AATGATACGGCGACCACCGAGATCTACACCTATTAAGTCGTCGGCAGCGTC |
| Illumina Reverse 5 | Illumina Index 1 (i7=N721) adapter | CAAGCAGAAGACGGCATACGAGATGCAGCGTAGTCTCGTGGGCTCGG |
| Illumina Reverse 6 | Illumina Index 1 (i7=N722) adapter | CAAGCAGAAGACGGCATACGAGATCTGCGCATGTCTCGTGGGCTCGG |
| Illumina Reverse 7 | Illumina Index 1 (i7=N723) adapter | CAAGCAGAAGACGGCATACGAGATGAGCGCTAGTCTCGTGGGCTCGG |
| **Sequencing** | | |
| UMI seq primer | Read 1 custom sequencing primer | AGAGACAGGACTAGGCAGTGGTGTGACCGCAGAGT |

RACE-RepSeq, 5' Rapid Amplification of cDNA Ends - Repertoire Sequencing

## Reference


1. Pascal, V., Dupont, M., De Rouault, P., Rizzo, D., Rossille, D., Jeannet, R., Daix, T., François, B., Genebrier, S., Cornic, M., et al. (2023). Demultiplexing Ig repertoires by parallel mRNA/DNA sequencing shows major differential alterations in severe COVID-19. iScience *26*, 106260. https://doi.org/10.1016/j.isci.2023.106260.

**Table S2.**

**Basic information of the datasets generated by each sequencing technology.**

| Method | Sample | Raw reads | Mean raw reads length (nt) | Mean raw reads Phred score | Reads after preprocessing | Productive IH V(D)J reads/contigs | Unique clones | Unique clonotypes |
|---|---|---|---|---|---|---|---|---|
| SMRT | T1 | 2,661,291 | 3,502 | 39.58 | 2,660,398 | 16,712 | 12,481 | 7,881 |
| | T2 | 2,901,891 | 3,524 | 39.57 | 2,900,863 | 19,635 | 16,190 | 11,361 |
| | T3 | 2,722,764 | 3,462 | 39.58 | 2,722,154 | 16,264 | 11,704 | 7,378 |
| RACE-RepSeq | T1 | 5,482,412 | 298 | 29.06 | 195,163 | 152,104 | 40,092 | 25,169 |
| | T2 | 5,360,553 | 299 | 29.02 | 160,057 | 124,571 | 40,820 | 25,951 |
| | T3 | 5,311,485 | 300 | 29.28 | 56,432 | 40,208 | 12,969 | 8,729 |
| ISRR | T1 | 28,350,239 | 151 | 38.74 | 368,696 | 24,555 | 7,549 | 5,618 |
| | T2 | 29,892,391 | 151 | 38.75 | 322,290 | 17,494 | 6,911 | 5,629 |
| | T3 | 32,632,349 | 151 | 38.82 | 339,830 | 19,686 | 6,219 | 4,804 |

ISRR: In silico, repertoire reconstruction from bulk RNA-seq data using TRUST4 algorithm;
RACE-RepSeq, 5' Rapid Amplification of cDNA Ends - Repertoire Sequencing;
SMRT, Single-Molecule Real-Time sequencing.

**Table S3**

**Descriptors and primer sequences for tracked clones, related to STAR Methods.**

| Descriptors of tracked clones | Isotype | Forward primer (5' to 3') | Reverse primer (5' to 3') |
|---|---|---|---|
| ***Clone sequences from RACE-RepSeq experiments*** | | | |
| IGHV3-53_J3_AIDDSNNVYAFHI (sample T1) | Gamma | GCCTCTGGATTCAGCGTCA | CTTGACCAGGCAGCCCAG |
| IGHV3-53_J3_ASDYSNYVYAFDV (sample T1) | Gamma | CAGCCTCTGGATTCAGCATCA | CTTGACCAGGCAGCCCAG |
| IGHV4-38-2_J3_ARHEESMAAIRVFDF (sample T1) | Gamma | CGCTGTCTCTGGTTTCTCCA | CTTGACCAGGCAGCCCAG |
| IGHV4-38-2_J1_ARFNGAIAPPG (sample T2) | Gamma | CGCTGTCTCTGGTTACTCCATC | CTTGACCAGGCAGCCCAG |
| IGHV4-39_J5_ARVYSGRWFDP (sample T2) | Alpha | CTGGTGACTCCATAAGTAGTGGT | GATGACCACGTTCCCATCTG |
| ***Clone sequences from ISRR*** | | | |
| IGHV4-4_J6_ARVVAADIEGDYGMDV (sample T1) | Alpha | GGTGGCTCCATCAGCAGTAG | GATGACCACGTTCCCATCTG |
| IGHV4-59_J5_AKEGSDYWALDWFDP (sample T1) | Alpha | GAGGCCCCATCAGTAAATCCTA | GATGACCACGTTCCCATCTG |
| IGHV3-11_J5_ARDGGRNSGWFYTYDS (sample T2) | Gamma | GCCTCTGGATTCACCTTCAG | CTTGACCAGGCAGCCCAG |
| IGHV3-11_J1_ARVGIVVEH (sample T3) | Gamma | GCCTCTGGATTCACCTTCAC | CTTGACCAGGCAGCCCAG |
| IGHV4-31_J6_ARVASNYGFWSGYRRGDYFYYYMDV (sample T3) | Gamma | CTGGTGGCTCCATCAGCAG | CTTGACCAGGCAGCCCAG |

ISRR: In silico, repertoire reconstruction from bulk RNA-seq data using TRUST4 algorithm;

RACE-RepSeq, 5' Rapid Amplification of cDNA Ends - Repertoire Sequencing

**Table S4.**

## Nucleotide sequences of tracked clones.

| Sequences of clones tracked in orthogonal validation |
|---|
| ***Clone sequences from RACE-RepSeq experiments*** |
| IGHV3-53_J3_AIDDSNNVYAFHI (sample T1) |
| GCCTCTGGATTCAGCGTCAATGCCACCAACATGACGTGGGTCCGCCAGGCTCCAGGGAAGGGGCTGGAGTGGGTCGGAGCTATCAATGGTGGTGGAGATACATACTACGGCGACTCCGTGAAGGGCCGATTCACCATCTCCAGAGACGATTCCGAGAACACCCTGTAT CTTCAAATGAACAGTGTGAGAGCCGAGGACACGGCCGTGTACTACTGTGCCATTGACGACAGTAACAACGTTTACGCTTTTCATATCTGGGGCCAAGGGACACTGGTCACCGTCTCTTTAGCCTCCACCAAGGGCCCATCGGTCTTCCCCCTGGCGCCCTGCTCCAGGAGC ACCTCCGAGAGCACAGCGGCCCTGGGCTGCCTGGTCAAG |
| IGHV3-53_J3_ASDYSNYVYAFDV (sample T1) |
| GCCTCTGGATTCAGCATCAATGCCACCAACATGACCTGGGTCCGCCAGGCTCCAGGGAAGGGGCTGGAGTGGGTCGCAGTTATCTATAGTGGTGGAGATACAGACTACGGCGACTCCGTGAAGGGCCGATTCACCATCTCCAGAGACGATTCCGAGAACACCCTGTATC TTCAAATGAACACTGTGAGAGCCGAGGACACGGCCGTATAYTATTGTGCCAGTGACTACAGTAACTACGTTTACGCTTTTGATGTCTGGGGCCAAGGGACACTGGTCACCGTCTCTTTAGCCTCCACCAAGGGCCCATCGGTCTTCCCCCTGGCGCCCTGCTCCAGGAGCA CCTCCGAGAGCACAGCGGCCCTGGGCTGCCTGGTCAAG |
| IGHV4-38-2_J3_ARHEESMAAIRVFDF (sample T1) |
| CGCTGTCTCTGGTTTCTCCATCAGCAGTTATTATTACTGGGCCTGGATCCGGCAGGCCCCAGGGAAGGGGCTGGAGTGGATTGGGAGTGTCTATCATGGTGGGACTTCCTTCTACAACCCGTCCCTCAGGAACCGTGTCACCATATCAGTAGACACGTCCAAGAACCAGT TTTCCCTGAGGCTGAGCTCTGTGACCGCCGCAGACACGGCCCTTTATTACTGTGCGAGACATGAGGAATCGATGGCTGCAATAAGAGTTTTTGATTTCTGGGGCCAAGGCACAATGATCACCGTCTCTTCAGCCTCCACCAAGGGCCCATCGGTCTTCCCCCTGGCACCCT CCTCCAAGAGCACCTCTGGGGGCACAGCGGCCCTGGGCTGCCTGGTCAAG |
| IGHV4-38-2_J1_ARFNGAIAPPG (sample T2) |
| CGCTGTCTCTGGTTACTCCATCACCACTGGTTACTACTGGGGCTGGATCCGGCAGCCCCCAGGGAAGGGGCTGGAGTGGATTGGGCTTATTTATCATAGTGGCAGCACCTACTACAACCCGTCCCTCAAGAGTCGTGTCACCATATCAGTGGACACGTCCAAGAACCATTT CTCCCTGAAGCTGAGTTCTGTGACCGCCGCAGACACGGCCATATATTACTGTGCGAGATTTAACGGTGCTATAGCACCACCTGGTGCTGGCCAGGGAACCCTGGTCATCGTCTCCTCAGCCTCCACCAAGGGCCCATCGGTCTTCCCCCTGGCACCCTCCTCCAAGAGCAC CTCTGGGGGCACAGCGGCCCTGGGCTGCCTGGTCAAG |
| IGHV4-39_J5_ARVYSGRWFDP (sample T2) |
| CTGGTGACTCCATAAGTAGTGGTAGTCACTACTGGGGCTGGATCCGCCAGCCCCCAGGGAAGGGGCTGGAGTGGATTGGGAGTATCAATTATAGTGGAGACACCCAGTACAACCCGTCCCTCAAGAGTCGAGTCACCATATCAGTAGACACGTCCAAGAACCAGTTCTC CCTGAAGCTGAGTTCTGTTACCGCCGCGGACACGGCCGTCTATCACTGTGCGAGAGTATACAGTGGACGGTGGTTCGACCCCTGGGGCCAGGGAACCCTGGTCACCGTCTCCTCAGCATCCCCGACCAGCCCCAAGGTCTTCCCGCTGAGCCTCTGCAGCACCCAGCCAG ATGGGAACGTGGTCATC |
| ***Clone sequences from ISRR*** |
| IGHV4-4_J6_ARVVAADIEGDYGMDV (sample T1) |
| GGTGGCTCCATCAGCAGTAGTAACTGGTGGAGTTGGGTCCGCCAGCCCCCAGGGAAGGGGCTGGAGTGGATTGGGGAAATCTATCATAGTGGGAGCACCAACTACAACCCGTCCCTCAAGAGTCGAGTCACCATATCAGTAGACAAGTCCAAGAACCAGTTCTCCCTG AAGCTGAGCTCTGTGACCGCCGCGGACACGGCCGTGTATTACTGTGCGAGAGTGGTCGCGGCCGACATAGAAGGTGACTACGGTATGGACGTCTGGGGCCAAGGGACCACGGTCACCGTCTCCTCAGCATCCCCGACCAGCCCCAAGGTCTTCCCGCTGAGCCTCTGCA GCACCCAGCCAGATGGGAACGTGGTCATC |
| IGHV4-59_J5_AKEGSDYWALDWFDP (sample T1) |
| GGAGGCCCCATCAGTAAATCCTACTGGAGCTGGATCCGGCAGCCCCCAGGGAGGGGACTGGAGTGGATTGGGTATATGTATTACAGTAGGAGCACCAACTACAACCCCTCCCTCAAGAGTCGAGTCACCATATCAGTGGACACGTCCAAGAACCAGTTCTCCCTGAAGC TGAGCTCTGTGACCGCCGCGGACACGGCCGTCTATTACTGTGCGAAAGAGGGTAGTGATTATTGGGCCCTGGACTGGTTCGACCCCTGGGGCCAGGGAACCCTGGTCACCGTCTCCTCAGCATCCCCGACCAGCCCCAAGGTCTTCCCGCTGAGCCTCTGCAGCACCCAG CCAGATGGGAACGTGGTCATC |
| IGHV3-11_J5_ARDGGRNSGWFYTYDS (sample T2) |
| GCCTCTGGATTCACCTTCAGTGACTACTACATGAGCTGGATCCGCCAGGCTCCAGGGAAGGGGCTGGAGTGGGTTTCATACATTAGTGGTAGTGGTAGTACCATATACTACGCAGACTCTGTGAAGGGCCGATTCACCATTTCCAGGGACAACGCCAAGAAGTCAGTGT TTCTGCAATTGAACAGCCTGAGAGCCGCGGACACGGCTGTTTATTACTGTGCGAGAGATGGGGGGCGGAACAGTGGCTGGTTCTACACGTATGACTCTTGGGGCCAGGGAACCCTGGTCAGCGTCTCCTCAGCCTCCACCAAGGGCCCATCGGTCTTCCCCCTGGCACC CTCCTCCAAGAGCACCTCTGGGGGCACAGCGGCCCTGGGCTGCCTGGTCAAG |
| IGHV3-11_J1_ARVGIVVEH (sample T3) |
| CTGGTGGCTCCATCAGCAGTGGTGGTTACTACTGGAGCTGGATCCGCCAGCCCCCAGGGAAGGGCCTGGAGTGGATTGGGTACATCTATTACAGTGGGAGCACCTACTACAACCCGTCCCTCAAGAGTCGAGTCACCATATCAGTAGACACGTCCAAGAACCAGTTCTC CCTGAAGCTGAGCTCTGTGACCGCCGCGGACACGGCCGTTTATTACTGTGCGAGAGTGGCATCAAATTACGGGTTTTGGAGTGGTTATCGAAGGGGTGACTACTTCTACTACTACATGGACGTCTGGGGCAAAGGGACCACGGTCACCGTCTCCTCAGCCTCCACCAAG GGCCCATCGGTCTTCCCCCTGGCACCCTCCTCCAAGAGCACCTCTGGGGGCACAGCGGCCCTGGGCTGCCTGGTCAAG |
| IGHV4-31_J6_ARVASNYGFWSGYRRGDYFYYYMDV (sample T3) |
| GCCTCTGGATTCACCTTCACAAATTACTACATCAGTTGGATCCGTCAGGCTCCAGGGAAAGGGCTGGAGTGGGTTTCACACATTAATGGTGGTAGTAATGTCATATATTACGCAGACTCTGTGAAGGGCCGATTCACCATCTCCAGGGACAACGCCAAGAATTCAGTGTA TCTGCAAATGAACAGCCTGAGAGCCGAAGACACGGCCGTTTATTACTGTGCGAGAGTGGGCATAGTGGTGGAGCACTGGGGCCAGGGAACCCTAGTCACCGTCTCCTCAGCCTCCACCAAGGGCCCATCGGTCTTCCCCCTGGCACCCTCCTCCAAGAGCACCTCTGGG GGCACAGCGGCCCTGGGCTGCCTGGTCAAG |

ISRR: In silico, repertoire reconstruction from bulk RNA-seq data using TRUST4 algorithm;

RACE-RepSeq, 5' Rapid Amplification of cDNA Ends - Repertoire Sequencing
